# Illustration of the Literature of Quackery

**Published:** 1874-09-15

**Authors:** 


### ILLUSTRATION OF THE LITERATURE OF QUACKERY.

THE August number of the Chicago *Pharmacist* contains the following editorial comments on the address on this subject delivered by Henry Gibbons, M. D., before the California State Medical Society, and which they republish in full from the *Pacific Medical and Surgical Journal:*

We call the attention of our readers to the address, so that they may view the topic through the spectacles of a medical editor, teacher and practitioner. As far as Dr. Gibbons has "illustrated" the subject, just so far it is good and truthful; but we sincerely regret that so fearless and able a writer as the author of this essay should have omitted mentioning the part the medical profession and its press play in the drama of Quackery.

We claim that the medical profession of this country is to a great extent responsible for the existing evil, and, to substantiate our charge, we will enumerate, first, the prescribing of nostrums by the medical profession, the composition of which they have not the remotest knowledge of; secondly, the giving of certificates of merit to the manufacturers of nostrums; thirdly, that the medical press of this country, with but few laudable exceptions, assist the use and sale of nostrums by inserting into the pages of the reading matter (styling it usually selected matter), month after month, cases of diseases which have been successfully treated by the use of this or that nostrum. These cases are written up by some "medical hireling," in the employ of these nostrum compounders, and the medical journals receive a money consideration for their insertion.

To prove the first charge we have made, it is only necessary to consult the prescription file of any dispensing pharmacy, where can be found prescriptions from regular graduated physicians, for such nostrums as McMunn's Elixir of Opium, Bromo Chloralum, Elixir Iodo Bromide of Calcium Compound, Cincho-Quinine, &c.

Secondly, on examining the wrappers attached to these nostrums, the names of physicians will be found endorsing the merits and the composition, the latter of which they are certainly ignorant of.

Thirdly, the medical press of this country has become, with *few* honorable exceptions, the aider and abettor of this species of fraud, by inserting among the reading matter paid "puffs," which seem to the uninitiated medical practitioner as *bona fide* reported cases of disease, successfully combatted by the means of this or that nostrum, when in reality it is written up by the man "Friday" of the nostrum houses, and by them sent to the different medical journals from month to month, paying a consideration for each insertion.

That this aiding of Quackery is not confined to the "little fish" of the profession, we will prove by referring to the present President of the American Medical Association, Wm. Bowling, M. D., of Nashville, Tenn., who is editor and proprietor of the Nashville *Journal of Medicine and Surgery*, late President of the meeting of American Medical Editors, and Emeritus Professor of the Theory and Practice of Medicine, in the Medical Department of the University of Nashville.

This gentleman, whom we have alluded to as holding these different prominent positions in the medical profession, inserted in the June number of his journal a "puff" for the well-known nostrum, Cincho-Quinine.

For this insertion he received, no doubt, the usual compensation from the manufacturers of this nostrum. Now we ask, what prompts men in such positions to sell themselves for such base purposes? It is certainly unprofessional, and cannot be styled honorable, for one who professes to be a leader of the science of medicine, and be guilty of such an offense against its progress.

Dr. Bowling and other editorial colleagues, who are guilty of this transgression, certainly cannot claim ignorance on this subject. How does this agree with Dr. Bowling's questions on professional ethics, published in his journal for July? Did the Doctor in his annual address, as President of the American Medical Editors' Association, touch upon the subject of advertising nostrums by the medical press? The address is said to have been both "suggestive and timely," or did it only refer to the more important medical topic, "Katy did, and Katy didn't?" Come, Doctor, step to the front, tell your readers you have been "naughty" for "filthy lucre's" sake, and you have thought the matter all over, and promise for the future to mend your evil ways. It shall not be said that Dr. Bowling "*knew the right, yet still the wrong pursued.*"

